# Control of cortical neuronal migration by glutamate and GABA

**DOI:** 10.3389/fncel.2015.00004

**Published:** 2015-01-30

**Authors:** Heiko J. Luhmann, A. Fukuda, W. Kilb

**Affiliations:** ^1^Institute of Physiology, University Medical Center of the Johannes Gutenberg UniversityMainz, Germany; ^2^Department of Neurophysiology, Hamamatsu University School of MedicineHamamatsu, Shizuoka, Japan

**Keywords:** neuronal migration, cerebral cortex, GABA, glutamate, neuronal migration disorders

## Abstract

Neuronal migration in the cortex is controlled by the paracrine action of the classical neurotransmitters glutamate and GABA. Glutamate controls radial migration of pyramidal neurons by acting primarily on NMDA receptors and regulates tangential migration of inhibitory interneurons by activating non-NMDA and NMDA receptors. GABA, acting on ionotropic GABA_A_-rho and GABA_A_ receptors, has a dichotomic action on radially migrating neurons by acting as a GO signal in lower layers and as a STOP signal in upper cortical plate (CP), respectively. Metabotropic GABA_B_ receptors promote radial migration into the CP and tangential migration of interneurons. Besides GABA, the endogenous GABAergic agonist taurine is a relevant agonist controlling radial migration. To a smaller extent glycine receptor activation can also influence radial and tangential migration. Activation of glutamate and GABA receptors causes increases in intracellular Ca^2+^ transients, which promote neuronal migration by acting on the cytoskeleton. Pharmacological or genetic manipulation of glutamate or GABA receptors during early corticogenesis induce heterotopic cell clusters in upper layers and loss of cortical lamination, i.e., neuronal migration disorders which can be associated with neurological or neuropsychiatric diseases. The pivotal role of NMDA and ionotropic GABA receptors in cortical neuronal migration is of major clinical relevance, since a number of drugs acting on these receptors (e.g., anti-epileptics, anesthetics, alcohol) may disturb the normal migration pattern when present during early corticogenesis.

## Introduction

During early brain development, mostly during embryonic phases and in some species also during early postnatal periods, newly generated neurons must migrate from their site of origin to their final target in a distinct brain area and a certain subregion, e.g., a specific layer at a certain site of the cerebral cortex. The distances that neurons must travel depend on the cell type and on the species and range from a few hundred micrometers to many millimeters. One exciting question in neuroscience is how newly generated neurons find their correct way to their final position. Over the last two decades we learned that the neuronal migration process is controlled by a number of different mechanisms. Transcription factors control the identity and the laminar position of developing neurons (for review, Kwan et al., 2012). Chemical cues, e.g., semaphorines and ephrins, are expressed as gradients in the brain and serve as attracting or repelling signals for migrating cells (Bagnard et al., 2001; Holmberg et al., 2006; Zimmer et al., 2008; Sentürk et al., 2011). In some brain regions the vertical fibers of radial glial cells act as chemico-mechanical guiding structures for migrating neurons (for review, Huang, 2009). Receptors activated by neurotransmitters or certain molecules, e.g., the extracellular matrix protein reelin, act as GO or STOP signals in neuronal migration (Huang, 2009).

This review will focus on the role of the two classical neurotransmitter systems glutamate and GABA in neuronal migration of cortical neurons. After briefly describing the different modes of neuronal migration and differences in the migration process between glutamatergic and GABAergic neurons, our current knowledge on the function of glutamate and GABA receptors in neuronal migration will be reviewed. Finally, we will also shortly address the putative role of glycine receptors in neuronal migration. Since the receptors of both transmitters are the target of numerous drugs acting for example as anesthetics or anti-epileptics, pathophysiological perturbations of the migration process by unwanted side effects of these drugs acting on glutamate and GABA receptors during early brain development will be also discussed.

For comprehensive overviews on the molecular and cellular mechanisms of neuronal migration the interested reader is referred to reviews by Ayala et al. (2007), Valiente and Marín (2010) and the review by David. J. Price on “Neuronal migration in the cerebral cortex” in this issue. A summary on neocortical layer formation, the timing of projection and interneuron migration and a comparison between rats and mice is given in the reviews by Kwan et al. (2012) and Tanaka and Nakajima (2012). An update on the early development of the human cerebral cortex is given by Bystron et al. (2008).

## Modes of neuronal migration

**Figure 1:**
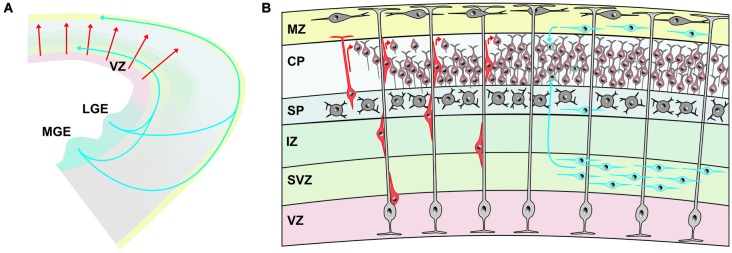
**Origin and migratory pathways of glutamatergic and GABAergic neurons. (A)** Schematic diagram illustrating migration pathway of the majority of glutamatergic neurons, originating in the ventricular zone (VZ) of the pallium and radially migrating into the developing cerebral cortex (red arrows). The majority of GABAergic neurons are generated in the medial (MGE) and lateral gangionic eminence (LGE) and reach their final position by tangential migration via deep pathways and superficial cortical layers. **(B)** Glutamatergic neurons (marked in different shades of red) are generated in the VZ and migrate radially either by somal translocation or, at later phases, by locomotion along radial glial cells (light gray). Upon reaching the marginal zone (MZ) they detach and align on top of previously generated neurons of the cortical plate (CP), generating the “inside first—outside last” pattern of the cerebral cortex. The majority of GABAergic neurons (marked in different shades of blue) reach the cortex via tangential migration in the deep pathway within the subventricular zone (SVZ) or the superficial pathway in the MZ. Some GABAergic interneurons travel also within the subplate (SP).

Different modes of neuronal migration have been described (Figure 1). In 1972, Pasko Rakic published a seminal paper on the “Mode of cell migration to the superficial layers of fetal monkey neocortex” and described the **radial migration** of immature neocortical neurons along the vertical fibers of radial glial cells (Rakic, 1972). Rakic postulated the existence of a “strong surface affinity” between the radial glial fibers and the migrating neuron and suggested that this “developmental mechanism … would allow for the vertical cell columns of adult neocortex” (Rakic, 1972). The radial unit hypothesis of the cerebral cortex was born. The radial glia-dependent locomotion is the dominant migration mode of newborn pyramidal, glutamatergic neurons in the hippocampus and cerebral cortex and also represents the central mechanism for the “inside first—outside last” developmental pattern of the cerebral cortex (neurons marked in red in Figure 1B; Nadarajah et al., 2003). Like building a house, the oldest neurons form the lowest layer 6 and subsequently generated neurons form layers 5, 4, 3 and finally layer 2. This inside-out layering also means that radially migrating neurons must pass beyond their predecessors before reaching their final position in the newly generated cortical layer, which they form (for review, Cooper, 2008). Recently Le Magueresse et al. (2012) described with time-lapse 2-photon microscopy in acute brain slice preparations of the neonatal mouse a new type of radial migration of subventricular zone (SVZ)-generated neurons along astrocytes lining blood vessels, which does not depend on radial glial cells.

A different mode of neuronal migration, which is independent of glial guiding fibers, is the **somal translocation** (Nadarajah et al., 2003; for review, Cooper, 2008). Somal translocation is smoother and faster than glia-guided radial migration. Here a leading coiled process extends into the marginal zone (MZ) and is anchored to the basement membrane or to the extracellular matrix. The soma moves upward in a spring-like manner by rapidly shortening the leading process. It seems likely that glia-independent somal translocation and glia-dependent locomotion depend on different cytoskeletal machinery and motors and thereby are regulated by different processes.

In contrast to the radial migration of pyramidal cells, neocortical GABAergic interneurons show a **tangential migration** pattern throughout the developing telencephalon (de Carlos et al., 1996; for review, Marín, 2013). Inhibitory interneurons migrate tangentially over long distances by generating a leading process, which detects chemical cues in the extracellular environment, and subsequent movement of the nucleus towards to the branching point (nucleokinesis). Recent observations in slice cultures of the mouse embryonic brain indicate that endothelial cells may guide tangential migration (Won et al., 2013) and that tangential migration in the MZ is controlled by meningeal vessels (Borrell and Marín, 2006). The molecular mechanism of this blood vessel-guided migration to the cortex are not known, but neurotrophic factors such as brain-derived neurotrophic factor (BDNF) and glial cell line-derived neurotrophic factor (GDNF) may be involved (Le Magueresse et al., 2012). Meninges affect tangential migration in the MZ via secretion of the chemokine CXCL12 which activates CXCR4 receptors (Borrell and Marín, 2006). This type of migration may become reactivated in the adult brain under pathophysiological conditions, e.g., stroke, when SVZ-generated neuroblasts are guided to the peri-infarct zone by blood vessels (Kojima et al., 2010).

Finally, so-called **random walk migration** has been described for medial ganglionic eminence (MGE)-derived cortical interneurons in the MZ of flat-mount cortices (Tanaka et al., 2009). Interneurons migrated tangentially over periods of up to 2 days in an unpredictable manner, often changing the rate and direction of migration. These results suggest that MGE-derived cortical interneurons, once arriving at the MZ, are released from regulation by guidance cues and initiate random walk movement (Tanaka et al., 2009).

In summary, radial migration, somal translocation and tangential migration are the dominant forms of neuronal migration in the developing cerebral cortex. It is not surprising that mutations affecting genes, which control these forms of migration may cause severe brain malformations, which are generally categorized as neuronal migration disorders and which are often associated with a spectrum of neurological and/or neuropsychiatric diseases (for review, Guerrini et al., 2008; Guerrini and Parrini, 2010).

## Migration of glutamatergic neurons

Neocortical glutamatergic neurons mostly follow a pure radial migration pattern and for them radial glial cells in the ventricular zone (VZ) fulfill two important and different functions in the embryonic cortex (Figure 1). On the one hand radial glial cells serve as progenitors and produce by asymmetric cell division neurons and astrocytes, on the other hand radial glial cells serve as migratory guides for the newly generated glutamatergic neurons. Radial glial cells produces neocortical pyramidal and layer 4 spiny stellate cells, which migrate to the cortical plate (CP), thereby forming in the “inside first—outside last” pattern the usually six-layered cerebral cortex. Sister glutamatergic neurons, which derive from the same mother cell, take up radially aligned positions in the cerebral cortex across layers and have a higher propensity to form unidirectional chemical synaptic connections with each other rather than with neighboring non-siblings (Yu et al., 2009). These data indicate that the columnar organization of the cerebral cortex may be determined to some extent by lineage (Noctor et al., 2001).

During early embryonic development, these glutamatergic neurons initially use somal translocation to migrate radially and then follow the vertical track along radial glial fibers for locomotion (Rakic, 1972). The extracellular matrix protein *reelin*, which is secreted from early born Cajal-Retzius neurons located in the MZ (for review, Kirischuk et al., 2014), controls this radial migration (for review, Valiente and Marín, 2010). Radial migration of single glutamatergic neurons does not occur continuously following a straightforward route, but rather shows phases of transient migratory arrest and even retrograde migration (Noctor et al., 2004). Gap junctions play important roles in the regulation of both proliferation and neuronal migration. Hemichannels formed by gap junctions mediate the spread of spontaneous intracellular Ca^2+^ waves across progenitor cells and provide dynamic adhesive contacts between migrating neurons and radial glial fibers (for review, Elias and Kriegstein, 2008). For glia-guided neuronal migration the connexins Cx26 and Cx43 are essential and in the mouse their deletion disrupts migration to the CP (Elias et al., 2007). For Cx43 it has been demonstrated that deletion of the C-terminal domain modifies neuronal migration (Cina et al., 2009).

Recent immunohistochemical data obtained in embryonic mice demonstrated one population of transient glutamatergic neurons, which is generated early (at embryonic day (E) 12.5) and migrates tangentially over long distances from their generation site at the pallial-subpallial boundary to the CP (Teissier et al., 2010). At birth, these early glutamatergic neurons homogeneously populate all neocortical areas, but subsequently die massively by apoptosis. At birth, about 50% of the dying neocortical neurons belong to this population of tangential migrating glutamatergic neurons (Teissier et al., 2010).

In summary, glutamatergic neurons use mostly radial migration along radial glial fibers and somal translocation to move from their site of generation in the VZ into the developing cerebral cortex.

## Migration of GABAergic neurons

In contrast to the large majority of the glutamatergic neurons, cortical GABAergic interneurons are at least in rodents generated in the subcortical telencephalon; in the lateral, medial, caudal and septal ganglionic eminence (LGE, MGE, CGE, and SGE, respectively; Figure 1A), to a minor extent also in the endopeduncular and preoptic area and also in the cortical SVZ (for review, Gelman and Marin, 2010) see also review by Wieland B. Huttner on “Neurogenesis in the developing cerebral cortex” in this issue. A subset of GABAergic neurons, which are 5-HT_3_ positive, are generated postnatally in the SVZ and migrate into numerous forebrain regions, including the cerebral cortex, striatum, and nucleus accumbens (Inta et al., 2008). The origin of GABAergic neocortical interneurons in higher mammals, including humans, remains controversial, although a recent publication indicate that also in these species a substantial proportion of interneurons originate from subcortical telencephalic eminences (Letinic et al., 2002; Ma et al., 2013).

The spatio-temporal expression of various transcription factors control the generation and identity of different types of cortical GABAergic interneurons at different developmental periods (for review, Butt et al., 2007; Jovanovic and Thomson, 2011). *Dlx1/2* and *Mash1* are extensively expressed in the ganglionic eminence and determine the GABAergic lineage. *Lhx6*, which is under the control of *Nkx2.1* and *Dlx5/6*, control the generation of parvalbumin- and somatostatin-immunoreactive interneurons, which are generated first in the ventral and dorsal area of the MGE, respectively (Wang et al., 2010). The later generation of vasoactive intestinal polypeptide (VIP) and cholecystokinine (CCK) expressing GABAergic interneurons in the CGE is controlled by the transcription factors *Nkx6.2* and *CoupTF1/2*. The spatio-temporal developmental profile of cortical GABAergic interneurons predicts their intrinsic electrophysiological properties and firing patterns in the mature cortex (Butt et al., 2005). Rapidly adapting firing properties can be observed in mature neuropeptide Y (NPY), reelin, calretinin and/or vasointestinal peptide expressing cortical interneurons, which are generated in the CGE. Rapidly adapting NPY-containing interneurons are also produced in the preoptic area (for review, Marín, 2013).

From their birth place in the ganglionic eminence forebrain GABAergic interneurons migrate tangentially in the MZ, SVZ or intermediate zone (IZ) to the developing cerebral cortex (for review, Marín, 2013). Tangential migration is controlled by the spatio-temporal expression of a number of chemical cues, acting as attracting or repelling signals. Semaphorines, expressed in the LGE, prevent the entry of migrating interneurons into this region and Ephrin EphA5/EphA4 receptors, expressed in the VZ, repel MGE-generated interneurons (for review, Marín, 2013). Tangential migration of cortical GABAergic interneurons is enhanced by the neurotrophic factors BDNF, NT-4, hepatocyte growth factor, and GDNF. On their way to the cortex, interneurons use specific routes or migratory streams (marked in blue in Figure 1B): (i) a superficial route in the MZ; (ii) a deep route in the IZ/SVZ; and (iii) a route in the subplate (SP). Using an *in situ* migration assay, Tanaka et al. (2003) observed that neocortical GABAergic interneurons initially migrate predominantly in the IZ/SVZ and then invade the CP and MZ by departing from the major migratory stream in the IZ/SVZ. Once arriving in the MZ GABAergic interneurons show random walk migration and disperse throughout the cortex (Tanaka et al., 2009). A subpopulation of GABAergic interneurons descend from the MZ to be distributed in the CP.

During their tangential migration process, neocortical GABAergic interneurons progressively acquire responsiveness to GABA. Combining *in vitro* patch-clamp recordings, neuropharmacological experiments and single-cell PCR in E14.5 mouse acute slices, Carlson and Yeh (2011) characterized the functional expression of GABA_A_ receptor subunits in tangentially migrating interneurons derived from the MGE. At this age, synapses have not yet formed and responsiveness to GABA reflect the functional expression of synaptic and extrasynaptic GABA_A_ receptors. Early migrating interneurons located close to the corticostriate juncture showed a robust expression of the alpha2 and alpha3 subunits. When entering the developing cortex, both subunits were still highly expressed and in addition alpha1 and gamma1-3 subunits were upregulated (Carlson and Yeh, 2011). The functional implications of the simultaneous activation of multiple GABA_A_ receptor isoforms and the upregulation of receptor isoforms with higher affinity to GABA in the migration process are not known and need to be elucidated.

Some experimental data indicate that migrating interneurons on their way to the cortex may move from one substrate to another, e.g., following specific axonal projections. Once they have reached their final cortical region, cortical GABAergic interneurons migrate radially to their final layer, which has been already formed by the radial migration of glutamatergic neurons. Thus, GABAergic interneurons invade their target layers after glutamatergic projection neurons have reached their final position. The mechanisms underlying this switch from tangential to radial migration are not completely understood. It may be that an intrinsic developmental program or connexins trigger the tangential-to-radial switch (for review, Marín, 2013). Elias et al. (2010) have demonstrated in embryonic rat brain slices including the MGE that this switch is controlled by Cx43 and depends on the adhesive properties and the C terminus of Cx43, but not on the Cx43 channel. These data indicate that the switch from tangential to radial migration depends on a gap junction-mediated interaction between migrating GABAergic interneurons and radial glia cells, similarly to the glia-dependent migration of glutamatergic neurons. In contrast, whereas reelin signaling is essential for proper radial migration of pyramidal neurons, layer acquisition of neocortical GABAergic interneurons does not depend on reelin, but rather on cues provided by projection neurons (Pla et al., 2006).

In summary, GABAergic interneurons migrate tangentially along specific streams from their site of origin in the subcortical telencephalon to their final neocortical site, where they then migrate radially to their final cortical layer.

## Role of glutamate in neuronal migration

The classical excitatory transmitter glutamate influences neuronal migration mainly by acting on two ionotropic receptors: (i) the NMDA receptor, a Ca^2+^-permeable subclass of glutamate receptor; (ii) the AMPA/kainate receptor, a usually Ca^2+^-impermeable glutamate receptor. Three (GluR1-3) of the four known subunits for AMPA receptors are expressed at prenatal stages in the developing cortex, while the GluR4 subunit appears only postnatally (Luján et al., 2005). Of the four subunits assembling kainate receptors, KA-2 and GluR5 and GluR6 are already expressed in the embryonic neocortex around E14 (Bahn et al., 1994). Functional NMDA receptors are composed from two NR1 and two NR2 subunits. NR1 and the highly Ca^2+^ permeable NR2B subunits are already expressed at early postnatal stages, while expression of NR2A emerges at postnatal stages in the neocortex (Luján et al., 2005). Functional NMDA receptors have been found on migrating glutamatergic and GABAergic interneurons (Behar et al., 1999; Soria and Valdeolmillos, 2002). Metabotropic glutamate receptors, in particular mGlu1 and mGlu5, are also already expressed in the immature neocortex (López-Bendito et al., 2002a). A direct modulation of neuronal migration by NMDA receptors has been initially described by Komuro and Rakic for granule cells of the developing mouse cerebellum *in vitro*. Here, blockade of NMDA receptors by specific antagonists caused a slow-down of neuronal migration, whereas enhanced activation of NMDA receptors by removal of magnesium from the extracellular milieu or by application of the cotransmitter glycine accelerated cell movement (Komuro and Rakic, 1993).

**Figure 2:**
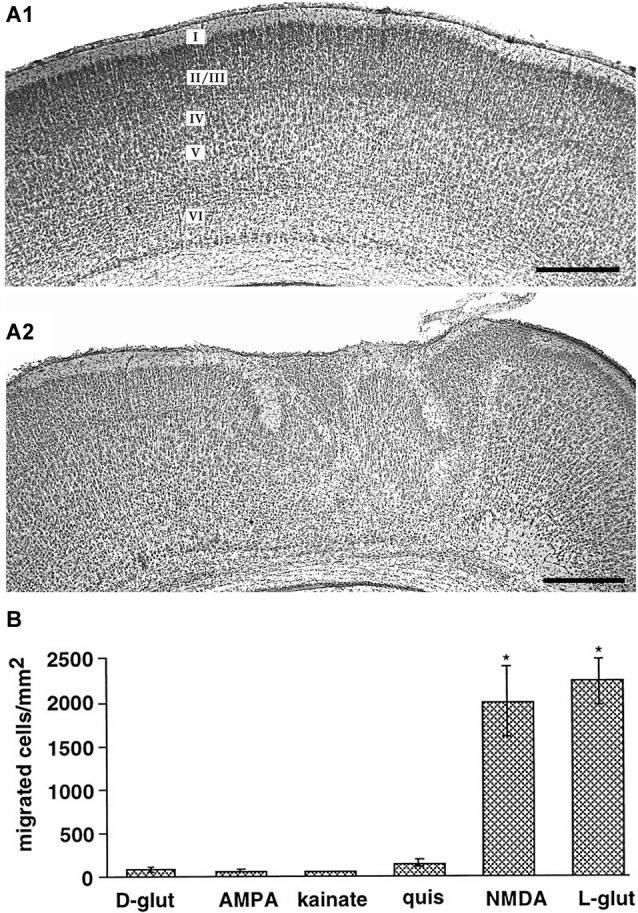
**Role of ionotropic glutamate receptors on radial migration *in vivo* and *in vitro*. (A)** Digital photographs of Nissl-stained coronal sections from a P7 rat that received at P0 on the cortical surface an Elvax implant containing DMSO (**A1**, control) or an implant loaded with the NMDA antagonist MK801 **(A2)**. Note abnormal cortical architecture and heterotopia in upper layers of the MK801-treated rat. **(B)** NMDA receptors mediate glutamate-induced migration of dissociated embryonic cortical cells *in vitro*. Reproduced with permission from Reiprich et al. (2005) **(A)** and Behar et al. (1999) **(B)**. Scale bars in **(A)** correspond to 500 µm.

Various *in vitro* studies using different models of cortical neuronal migration indicate that NMDA receptors also control radial neuronal migration in the cerebral cortex. In cell dissociates of murine embryonic cortical cells and cortical slice cultures, Behar et al. (1999) demonstrated that glutamate is a potent chemoattractant. Only activation of NMDA receptors, but not other ionotropic glutamate receptors, stimulated radial migration of immature neurons out of the cortical VZ/SVZ and application of NMDA antagonists blocked migration (Figure 2B; Behar et al., 1999). Inhibition of NMDA receptors using either MK801 or APV, attenuated radial migration in rat tissue explants *in vitro* (Hirai et al., 1999). In contrast to these observations, which suggest a promigratory effect of NMDA receptors, a massive stimulation of NMDA receptors led to migratory arrest in cultured cerebral neurons (Kihara et al., 2002), indicating that only physiological levels of NMDA receptor activation may be a prerequisite for a promigratory stimulus. The observations that (i) Mg^2+^ depletion enhances migration (Behar et al., 1999); (ii) overexpression of the Mg^2+^-sensitive NR2B subunit increases migration (Tárnok et al., 2008); and (iii) the NR2B subtype specific antagonist ifenprodil hinder migration of cerebellar neurons (Mancini and Atchison, 2007), all indicate that Mg^2+^ sensitive NMDA receptors are involved in regulating neuronal migration. It has been also suggested that depolarized membrane potentials of migrating neurons contribute to the relative Mg^2+^ insensitivity of the NMDA receptor-mediated effects (Gerber et al., 2010).

Also in tangentially migrating neocortical interneurons an inhibition of NMDA and AMPA receptors impedes migration (Bortone and Polleux, 2009), in accordance with the functional expression of NMDA and non-NMDA ionotropic glutamate receptors in migrating interneurons (Soria and Valdeolmillos, 2002). Unfortunately this study does not allow to discriminate whether AMPA and/or NMDA receptors affect migration. However, at least for mouse hippocampal interneurons it has been demonstrated that AMPA, but not NMDA receptors, influence radial migration (Manent et al., 2006). Therefore further analysis whether AMPA receptors are involved in the tangential migration of neocortical interneurons is required to elucidate if this is a common feature of interneuronal tangential migration.

In neurospheres it has been demonstrated that the early phases of neural progenitor cell migration strictly depend on AMPA receptors (Jansson et al., 2013). However, it is currently unclear whether AMPA receptors also contribute early phases of radial and/or tangential neuronal migration under *in vivo* conditions.

Further evidences for a role of glutamate in migration of neocortical neurons came from *in vivo* studies. Using intracerebral injections of ibotenate, an agonist of NMDA receptors and glutamatergic metabotropic receptors, Marret et al. (1996) demonstrated in the hamster by neuropharmacological experiments that activation of NMDA receptors caused a wide spectrum of abnormal neuronal migration patterns in the cerebral cortex *in vivo*. Golden hamsters were chosen for these studies because compared to mice and rats the cortex in hamsters is very immature at birth. While low doses of ibotenate produced mainly intracortical heterotopias and molecular layer ectopias, indicating an disturbed termination of migration, high ibotenate doses led mainly to periventricular and subcortical heterotopias, suggesting that they affected migratory onset (Marret et al., 1996). These migration defects could be attributed to both migration arrest and unsufficient termination of migration (Takano et al., 2004). Using sustained-release polymer Elvax implants (Smith et al., 1995) containing MK801 to deliver this NMDA antagonist focally to the cortical surface, Reiprich et al. (2005) could demonstrate that a local and transient NMDA receptor blockade in the somatosensory cortex of newborn rats *in vivo* produces structural and functional alterations in the cortical region underlying the implant (Figure 2A). MK801-treated animals showed disturbances in the cortical lamination and heterotopic cell clusters in the upper layers.

Complete knockout of NR1, an essential subunit of NMDA receptors, has no effect on the early migration pattern of neocortical neurons in the fetal mouse brain, but mice die at birth due to respiratory problems (Messersmith et al., 1997). A restricted knockout of NR1 in excitatory neocortical neurons (CxNR1KO) led to only slight changes in the neocortical organization, like a disordered barrel cortex, without gross anatomical disturbances reminiscent of cortical migration disorders (Iwasato et al., 2000), but in these animals residual amounts of functional NMDA receptors may be present during prenatal development. The function of NR1 in neuronal migration may be also compensated by other mechanisms in CxNR1KO. On the other hand, in chimeric mice transfected with NR1-deficient stem cells, neurons without functional NMDA receptors show a normal distribution within the hippocampus, indicating that NMDA receptors on neuronal membranes itself may be dispensable for correct radial migration (Maskos and McKay, 2003).

Thus no final conclusion on the role of NMDA receptors for migration can currently be given. While pharmacological *in vivo* and *in vitro* experiments strongly suggest an important role of NMDA receptors for radial migration, the observation that neurons lacking functional NMDA receptors show adequate migration questions this conclusion. These conflicting results may either indicate that the NMDA receptor dependent effects are mediated by non-neuronal target structures like glial cells or that compensatory mechanisms may counteract the lack of functional NMDA receptors.

The source of extracellular glutamate controlling neuronal migration is not completely known. *In vitro* studies on hippocampal organotypic slice co-culture assays from munc18-1 knockout mice, in which vesicular transmitter release is deleted, indicate that glutamate and also GABA is released in a SNARE-independent manner and both transmitters control neuronal migration via a paracrine action (Manent et al., 2005). Another mechanism of extracellular transmitter control are transporters. Glutamate uptake by transporters expressed in astrocytes set extracellular glutamate levels. The expression of glutamate transporters is relatively low in immature rodent hippocampus and increases during early postnatal development (Thomas et al., 2011), suggesting that extracellular concentrations of glutamate may be higher during early corticogenesis when neuronal migration occurs. However, extracellular space is also larger during early development (for review, Syková, 2004), therefore overall extracellular transmitter concentrations in the young brain may be not so much higher than in adult. Furthermore, inhibition of glutamate uptake enhances migration (Komuro and Rakic, 1993), which indicates that glutamate is sequestered rather than released in the vicinity of migration neurons. Related to the glutamatergic system, it has been demonstrated in the cerebellum that glutamate activates Bergmann glial cells to produce and release d-serine, which potentiates glutamate actions on NMDA receptors and enhances neuronal migration of cerebellar granule neurons (Kim et al., 2005).

**Figure 3:**
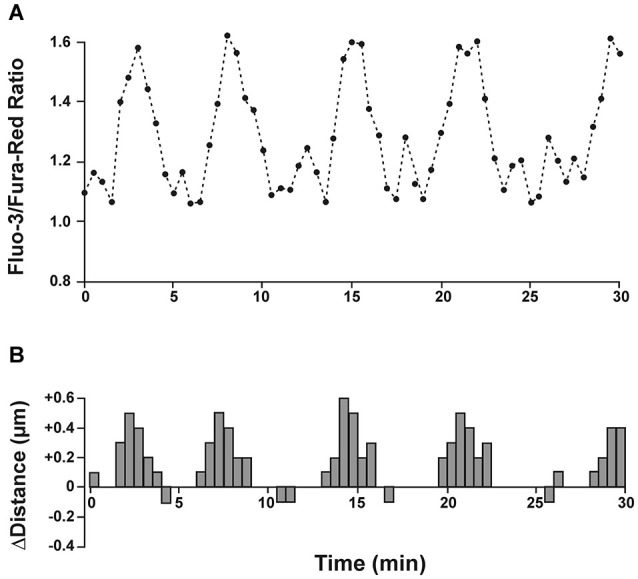
**Spontaneous intracellular calcium fluctuations correlate with migration speed and direction. (A)** Granule cells in cerebellar microexplant cultures were loaded with a mixture of the two calcium indicators Fluo-3 and Fura-Red. Upward deflections in Fluo-3/Fura-Red ratio indicate intracellular calcium rise and downward deflections represent calcium decrease. **(B)** Distance and direction of the same cell as in A. During a recording period of 30 min the migrating neuron exhibited 5 cycles of saltatory movements, which closely correlated with transient intracellular calcium changes. Modified and reproduced with permission from Komuro and Rakic (1996).

The downstream molecular mechanisms how glutamate controls neuronal migration are not completely understood, but an appropriate increase in the intracellular Ca^2+^ level is pivotal (for review, Komuro and Kumada, 2005; Zheng and Poo, 2007). Elegant experiments performed on migrating cerebellar neurons *in vitro* demonstrated that migratory and resting phases were directly correlated to elevated and resting Ca^2+^ concentrations, respectively (Figure 3; Komuro and Rakic, 1996). In addition, this study demonstrates that the amplitude of Ca^2+^ transients is directly correlated to the rate of saltatory cell movements. Disappearance of these Ca^2+^ transients triggered the completion of cerebellar granule cell migration (Kumada and Komuro, 2004). In an interesting experiment Fahrion et al. (2012) were able to rescue methylmercury-induced migratory arrest of murine cerebellar neurons by restoring the frequency of Ca^2+^ transients to control levels. Further support for a pivotal role of intracellular Ca^2+^ in controlling neuronal migration comes from experiments in which the Ca^2+^ chelator BAPTA inhibited radial migration in murine cerebellar (Komuro and Rakic, 1993) and murine neocortical cells (Hirai et al., 1999). Interestingly, soma translocation in migrating GABAergic interneurons depend on the occurrence of non-symmetrical Ca^2+^ signals, with larger Ca^2+^ transients observed toward the direction of migration (Moya and Valdeolmillos, 2004). On the other hand, a tonic Ca^2+^ increase arrested motility in the absence of Ca^2+^ transients (Komuro and Rakic, 1996). These data demonstrate that fluctuations in the intracellular Ca^2+^ concentration within a physiological range control normal neuronal migration.

The Ca^2+^ transients can interfere with the organization of the cytoskeleton via an activation of Ca^2+^ dependent kinases, like Ca^2+^-calmodulin kinases II or doublecortin (DCX)-like kinases (Kumada and Komuro, 2004; Koizumi et al., 2006). Accordingly, inactivation of either DCX or the DCX-like kinase by shRNA slowed radial and tangential neuronal migration (Friocourt et al., 2007, see also review by Fiona Francis on “*The roles of DCX in cortical development*” in this issue). In addition, a Ca^2+^ increase also activates Lis1-dependet rho-kinases, which are involved in connecting the microtubules in a Clip170 dependent manner to the actin cytoskeleton and dynein motor complexes (Kholmanskikh et al., 2006, see also review by Emilie Pacary on “*Role of RhoGTPases in cerebral cortex development*” in this issue). Interestingly, mutations in Lis1 and DCX have been directly linked to human neocortical migration disorders (Gleeson and Walsh, 2000).

In summary, there is compelling evidence that glutamate controls radial migration of glutamatergic neurons, most probably by acting on NMDA receptors. The mechanisms of the glutamate effect on tangential migration of GABAergic interneurons is less established and here AMPA receptors are more relevant.

## Role of GABA and taurine in neuronal migration

The classical inhibitory neurotransmitter GABA is important in controlling neuronal migration via ionotropic GABA_A_ and metabotropic GABA_B_ receptors (Manent and Represa, 2007). GABA_A_ receptors are heteropentamers compiled from in total 19 subunits, divided into eight groups, while GABA_B_ receptors are heterodimers co-assembled from the GABA_B2_ subunit with one of the two isoforms of the GABA_B1_ subunit (for a detailed review, see Farrant and Kaila, 2007; Ulrich and Bettler, 2007). Several GABA receptor subunits are abundantly expressed during early cortical development. At E14 the GABA_A_ receptor subunits α_2_, α_3_, α_4_, β_1_ and γ_1_ are expressed, with α_3_ expressed at particular high levels during prenatal development (Laurie et al., 1992). Accordingly, GABA_A_ receptor mediated currents are observed already in proliferative neuroblasts and early postmitotic neurons (LoTurco et al., 1995; Owens et al., 1999). In line with the paucity of α_1_ and γ_2_ expression, immature cortical neurons show GABA_A_ receptor mediated currents with slow kinetics and little desensitization, high GABA affinity and lack of synaptic GABAergic currents before they terminate migration in the CP (Owens et al., 1999). In addition to this classical GABA_A_ receptor, ρ subunit containing GABA_A_-rho receptors, characterized by an exceptionally high GABA affinity and little desensitization, are found in the SVZ, while they are lacking in CP neurons (Denter et al., 2010). GABA_B1_ and GABA_B2_ subunits are expressed throughout all neocortical lamia during early stages of cortical development (López-Bendito et al., 2002b). Interestingly tangentially migrating neurons express only GABA_B1_ subunits and should thus lack functional GABA_B_ receptors (López-Bendito et al., 2002b). Finally, it is important to consider that immature neocortical neurons show a high ratio in the expression of NKCC1 to KCC2, which renders GABA_A_ mediated responses depolarizing (Yamada et al., 2004).

**Figure 4:**
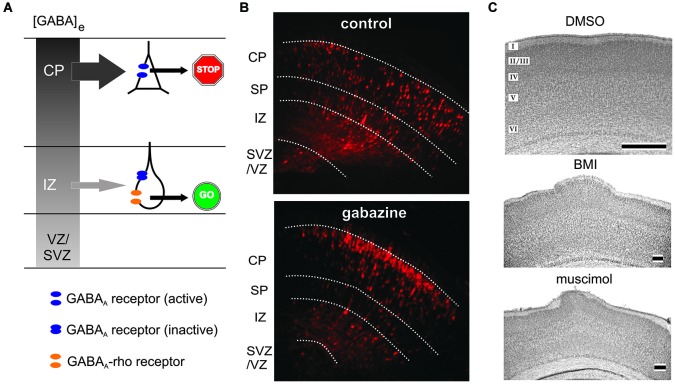
**Role of ionotropic GABA receptors on radial migration. (A)** Model of GABA_A_ and GABA_A_-rho receptor dependent radial migration in the neonatal cerebral cortex, which shows outside directed GABA gradient (gray colored gradient). In the IZ migrating neurons express functional GABA_A_ receptors (blue discs) and GABA_A_-rho receptors (orange discs), whereas in the CP migrating neurons express only functional GABA_A_ receptors. Due to the outside directed GABA gradient the low-affinity GABA_A_ receptors are only activated in the CP, while the lower GABA concentration in the IZ is sufficient to activate the high affinity GABA_A_-rho receptors. Activation of GABA_A_-rho receptors is necessary to support migration in the IZ (GO sign), while activation of GABA_A_ receptors contributes to termination of migration (STOP sign). **(B)** Blockade of GABA_A_ receptors with gabazine facilitates radial migration. Figures illustrate red fluorescent protein (RFP)-positive cells in control (left) and gabazine-treated (right) GAD67^GFP/GFP^ fetuses at E17.5, which were injected with gabazine at E14.5 immediately after the electroporation of the RFP vectors. **(C)** Digital photographs of Nissl-stained coronal sections from a P7 rat that received at P0 on the cortical surface an Elvax implant containing DMSO (top, control), the GABA_A_ antagonist bicuculline methiodide (middle, BMI) or the GABA_A_ agonist muscimol (bottom). Note upper layer heterotopia due to increased radial migration in BMI- and muscimol-treated animals. Scale bar in B, C middle and C bottom corresponds to 200 µm, in C top to 500 µm. Modified with permission from Denter et al. (2010) **(A)**, Furukawa et al. (2014) **(B)**, and Heck et al. (2007) **(C)**.

The implication of GABA receptors in the control of neuronal migration was first demonstrated by Behar et al. (1996), who could show by the use of a microchemotaxis chamber that neuronal migration of dissociated cortical neurons of embryonic rats is stimulated by low concentrations of GABA acting on GABA_A_/GABA_A_-rho and GABA_B_ receptors. Femtomolar concentrations of GABA induced chemotaxis (migration along a chemical gradient) and micromolar GABA initiated chemokinesis (increased random movement). In a subsequent study Behar et al. (1998) showed that 1–5 µM GABA stimulated the migration of GAD-expressing neurons in the CP, whereas 500 fM stimulated motility of GAD-expressing neurons in the VZ. In this study the authors also postulate that GABA can promote migration via G-protein activation, mediated by GABA_B_ receptors, and arrest migration via GABA_A_ receptor-mediated depolarization (Behar et al., 1998). Using organotypic neocortical slice cultures Behar et al. demonstrated that specific activation of the different GABA receptors modified different parts of neural migration to the CP (Behar et al., 2000): (i) GABA_A_/GABA_A_-rho receptor activation promoted neuronal migration from the VZ/SVZ to the IZ; (ii) GABA_B_ receptor controlled the migration from the IZ into the CP; and finally (iii) GABA_A_ receptor activation provided the stop signal to terminate migration in the upper CP (Behar et al., 2000). The two ionotropic GABA receptor subtypes, GABA_A_ and GABA_A_-rho receptors, play different roles in the control of radial migration (Figure 4A). In organotypic murine neocortical slice cultures application of bicuculline methiodide (BMI) facilitated neuronal migration, while the GABA_A_-rho specific antagonist TPMPA attenuated migration (Denter et al., 2010). These data add important information to the model on the role of GABA in neuronal migration as initially introduced by Behar et al. (2000). GABA_A_-rho receptors have a high affinity to GABA and are transiently expressed on migrating neurons in the IZ, where the extracellular GABA concentration at late embryonic stages is relatively low (Figure 4A). In the IZ, GABA acting primarily on GABA_A_-rho receptors acts as a GO signal for migrating neurons coming from the VZ/SVZ and passing through the IZ to the CP. Reaching the CP, migrating neurons no longer express functional GABA_A_-rho receptors. Due to the intracortical outside directed GABA gradient low-affinity GABA_A_ receptors are now activated and GABA serves as a STOP signal in the upper CP (Denter et al., 2010). Although this model is supported by a number of experimental studies, data on the existence of the outside directed GABA gradient are scare. Regional differences in the amplitudes of GABA_A_ receptor-mediated tonic currents suggest a gradient of endogenous GABA_A_ receptor agonists in embryonic murine cortex (Furukawa et al., 2014). However, detailed information about spatial gradients in extracellular GABA concentrations are currently not available (Bolteus et al., 2005). GABA imaging in brain slices using immobilized enzyme-linked photoanalysis may provide a possibility to demonstrate such a gradient (Morishima et al., 2010).

Blockade of GABA_B_ receptors in organotypic cultures of the rat brain led to an accumulation of tangentially migrating neurons in the VZ/SVZ of the rat neocortex (López-Bendito et al., 2003), suggesting a role of GABA_B_ receptors for final apposition of GABAergic interneurons. However since no functional GABA_B_ receptors are expressed on these neurons (López-Bendito et al., 2002b), this effect may be caused by GABA_B_ receptor-dependent effect on targets upstream from the migration tangential neurons itself (López-Bendito et al., 2002b).

A direct influence of GABA on the migration of cortical neurons has also be shown *in vivo*. Furukawa et al. (2014) demonstrated *in vivo* that continuous blockade of GABA_A_ receptors with the GABA_A_ antagonist gabazine (SR95531) during late embryonic stages accelerated radial migration in the murine neocortex (Figure 4B). In line with this, Elvax implants loaded with the GABA_A_ antagonist BMI or the agonist muscimol placed on the neocortical surface of newborn rats induced heterotopic cell clusters in upper layers and a loss of neocortical lamination, probably because of an overmigration from the loss of a stop signal (Figure 4C). Whereas BMI caused this effect by blocking GABA_A_ receptors, long-term application of muscimol induced a pronounced receptor desensitization, thereby also reducing GABA_A_ receptor function on migrating rat neurons (Heck et al., 2007). In accordance with the results of *in vitro* studies identifying the role of GABA_B_ receptors (Behar et al., 2001), *in utero* knockdown of GABA_B_ receptors using RNA interference techniques impaired radial migration of the affected pyramidal neuron progenitors in the rat neocortex (Bony et al., 2013). The superficial stream of tangentially migrating GABAergic interneurons in the MZ of neonatal mice is also impaired after inhibition of GABA_A_ receptors *in vivo* (Inada et al., 2011), demonstrating a direct influence of endogenous GABA also on tangential migration.

In summary, these reports demonstrate that ionotropic GABA_A_ and metabotropic GABA_B_ receptors are involved in the control of neuronal migration in the cortex.

One feature of the GABA receptors expressed on migrating neurons is their high GABA affinity, allowing them to sense even low ambient GABA concentrations. Microdialysis experiments in tangential neocortical slices revealed an extracellular GABA concentration of 25 nmol/l in the MZ of early postnatal rats (Qian et al., 2014), however, this experimental paradigm may underestimate the interstitial GABA concentration close to migrating neurons. Using GABAergic modulation of glutamate release Dvorzhak et al. (2010) suggested a juxtasynaptic GABA concentration of 250 nmol/l during early postnatal stages in the mouse, with a substantial developmental decrease during the first postnatal week. A substantially higher ambient GABA concentration of ~0.5 µmol/l was observed in the ganglionic eminence of mice using GABA_A_ receptor expressing sniffer cells as GABA sensors (Cuzon et al., 2006), which may be relevant for the tangential migration of GABAergic interneurons from this region.

It was suggested that tangentially migrating GABAergic neurons are a source for GABA (Manent et al., 2005). *In vitro* assays indicated that CP neurons itself secrete promigratory signals acting on GABA receptors and suggested that these signals may include GABA and/or taurine (Behar et al., 2001). In line with this, tangentially migrating neurons in GAD67-GFP knock-in mice had a substantially slower migration rate, which has been attributed to the lower extracellular GABA level in these animals (Inada et al., 2011). However, no obvious disorders in gross neocortical organization have been observed after complete blockade of GABA synthesis in GAD65/GAD67 knockout mice (Ji et al., 1999), indicating that other substances can act as GABAergic agonists during prenatal development.

**Figure 5:**
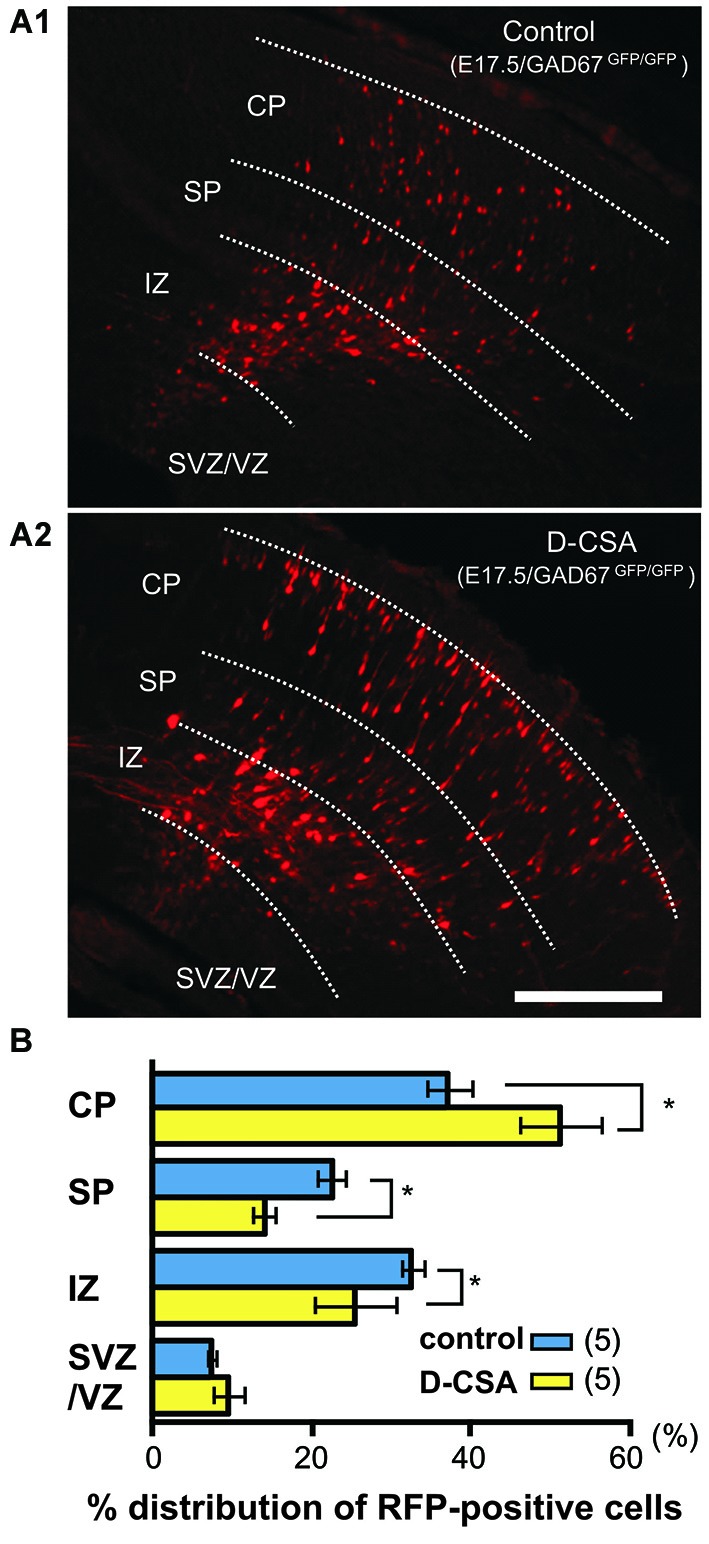
**Suppression of taurine synthesis by maternal D-cysteine sulfinic acid (D-CSA) i.p. administration accelerates radial migration. (A)** Distribution of RFP-labeled radially migrating neurons in cortical sections from GAD67^GFP/GFP^ fetuses at E17.5. Saline (control, **A1**) or D-CSA **(A2)** was administered to the mothers every12 h between E14 and E17. **(B)** Averaged proportions of radially migrating cells in the CP, SP, IZ, and VZ/VZ of cortical slices prepared from fetuses at E17.5 with (yellow bars) and without (blue bars, control) maternal D-CSA administration. Scale bar in A2: 200 µm. Modified with permission from Furukawa et al. (2014).

A recent study identified taurine, released by volume-sensitive anion channels, as an important agonist of GABA_A_ receptors directly influencing radial migration and its action was most apparent in the SP where taurine is most abundant (Figure 5). A decrease in ambient taurine, via pharmacological blockade of taurine synthesis, accelerated radial migration in the developing cerebral cortex. This effect was clearly mediated via GABA_A_ receptors and is more substantial in GAD67 deficient mice with reduced extracellular GABA levels (Furukawa et al., 2014). Thus ambient GABA is not negligible, although ambient taurine is a main endogenous agonist. In addition, it was recently shown that taurine inhibits KCC-2 activity via activating the with-no-lysine protein kinase 1 (WNK1) and downstream STE20/SPS1-related proline/alanine-rich kinase (SPAK)/oxidative stress response 1 (OSR1) signaling pathway (Inoue et al., 2012). Thereby it may also play a role in maintaining the depolarizing GABAergic responses required for a promigratory action (see below). Microdialysis experiments in the MZ of early postnatal rats revealed a taurine concentration of 33 µmol/l, which was substantially higher than the GABA concentration (Qian et al., 2014). Thus taurine must also be considered as an important endogenous agonist influencing migration via GABA receptors.

Various mechanisms of GABA release at early stages of corticogenesis have been suggested. GABAergic precursor cells may release GABA tonically in a Ca^2+^- and SNARE-independent manner. Blocking GABA_A_ receptors in hippocampal slice cultures from munc18-1-deficient mice, in which vesicular release is abolished, impairs neuronal migration, which supports the hypothesis that GABA is released in a non-canonical, paracrine manner (Manent et al., 2005). Candidates for a non-vesicular GABA release are GABA transporters (GATs), which take up GABA from the interstitial space. Whereas in the adult brain GAT-1 is mainly expressed in neurons and GAT-3 mainly in glial cells, in the immature cortex both GAT isoforms are expressed in astrocytes and neurons (for review, Kilb et al., 2013) and both transporters can release GABA by acting in reverse mode (for review, Kirischuk and Kilb, 2012). A non-vesicular release of GABA via reversal of GAT-1 has been demonstrated in tangentially migrating interneurons following glutamate induced activation of AMPA receptors and sodium influx (Poluch and König, 2002). In the MZ, GATs represent the major mechanism of GABA release and their operating mode is influenced by excitatory amino acid transporters (EAATs) via intracellular sodium signaling and/or cell depolarization (Unichenko et al., 2013), indicating that ambient glutamate and GABA levels are mutually dependent. As an alternative mechanism of GABA release, GABA could also be released via anion channels such as bestrophin-1 channel (Lee et al., 2010). However, no direct evidence of GABA release via volume-sensitive anion channels has yet been reported. In contrast, a recent study identified taurine, released by volume-sensitive anion channels, as an important agonist of GABA_A_ receptors (Figure 5; Furukawa et al., 2014).

In summary, non-synaptic release of GABA and taurine most probably is the main source of ambient GABAergic agonists that influence neuronal migration. However, the contribution of different release mechanisms for GABA and taurine to their ambient levels have not been determined.

The down-stream pathways of migration control by GABA and taurine are not fully understood. As for glutamate, GABA-induced intracellular Ca^2+^ transients are essential. Impairing GABA_A_ receptor dependent Ca^2+^ signals by Ca^2+^ chelators inhibits the chemotropic effect of GABA in cortical cells (Behar et al., 1996) and impedes tangential migration (Inada et al., 2011). These GABA induced Ca^2+^ transients are mediated by developmental changes in the expression of chloride transporters, leading to depolarizing GABAergic responses (for review, Ben-Ari et al., 2007; Kilb, 2012; Kaila, 2014; Luhmann et al., 2014). Whereas the chloride inward transporter NKCC1 is highly expressed in immature neurons, the efficacy of the chloride outward transporter KCC2 is low, and this imbalance in chloride transport causes a high intracellular concentration of chloride ions (Yamada et al., 2004). In accordance with this hypothesis, a pharmacological blockade of NKCC1 using bumetanide impairs tangential migration of murine GABAergic interneurons *in vivo* (Inada et al., 2011). This hypothesis was challenged by the observation, that premature expression of KCC2 by *in utero* expression at E17/18 causes no obvious migration deficits of rat neocortical neurons, while causing a hyperpolarizing shift in the chloride reversal potential of GABA-induced currents at early postnatal stages (Cancedda et al., 2007). This result is not too surprising, because ectopically expressed wild type KCC2 is not active in embryonic cerebral cortices and becomes functional only postnatally (Inoue et al., 2012). In addition, the *in utero* expression was performed at relatively late stages, so that a substantial part of radial migration to layer II/III was already accomplished until E21 (Cancedda et al., 2007). Indeed, ectopic expression of constitutive active KCC2 mutant at E15 lowered intracellular chloride concentrations, rendered hyperpolarizing GABA_A_ receptor mediated responses in postmitotic neurons and perturbed their radial migration (Inoue et al., 2012). In migrating murine interneurons the chloride outward transporter KCC2 increases in expression and becomes functional after they enter the cerebral cortex (Bortone and Polleux, 2009), resulting in a reduced intracellular chloride concentration. The consequent shift in GABAergic action from excitation to inhibition leads to a decrease in the frequency of spontaneous intracellular Ca^2+^ transients and terminates neuronal migration, thus turning GABA into a STOP signal for migrating interneurons (Bortone and Polleux, 2009). This scenario is supported by experimental data from Inoue et al. as mentioned above (Inoue et al., 2012).

In addition to a direct excitatory effect, depolarizing GABAergic responses are also involved in spontaneous activity patterns observed in neocortical networks during pre- and early postnatal development (for review, Khazipov and Luhmann, 2006; Allene and Cossart, 2010; Kilb et al., 2011). In a rat neocortical culture model de Lima et al. (2009) demonstrated a relationship between the expression of spontaneous synchronous network activity and neuronal migration. Although migrating interneurons did not participate in early cortical network activity, migration was terminated when interneurons became active in a synchronous network. These data indicate that synchronized GABA and also glutamate release during early network activity can terminate neuronal migration (de Lima et al., 2009).

In summary, GABA and the endogenous GABAergic agonist taurine have a strong impact on tangential and radial migration. These neurotransmitters have both, promigratory and migration-terminating actions, depending on the type of GABA receptor and the intracellular chloride concentration in the migrating neuron.

## Role of glycine in neuronal migration

Beside ionotropic GABA receptors, glycine receptors also have an influence on neuronal migration. As GABA_A_ and GABA_A_-rho receptors, glycine receptors are also transmitter-gated chloride channels, which upon activation by glycine or taurine mediate a depolarizing or even excitatory action in the immature cortex (Flint et al., 1998; Kilb et al., 2002, 2008). A functional expression of heteromeric glycine receptors, compiled from α2/β subunits, has already been described in various types of immature neurons, including putative migratory neurons in the IZ (Flint et al., 1998; Kilb et al., 2002, 2008; Okabe et al., 2004), whereas tangentially migrating neurons express α2 homomeric glycine receptors (Avila et al., 2013). It is therefore not surprising that an activation of glycine receptors also promoted radial neuronal migration as demonstrated in organotypic slice cultures from embryonic mouse cerebral cortex (Nimmervoll et al., 2011). However, as pharmacological inhibition of glycine receptors did not interfere with radial migration, Nimmervoll et al. (2011) suggest that glycine receptors do not contribute substantially to radial migration in the neocortex (see also Furukawa et al., 2014). In contrast, tangential migration of cortical interneurons was effectively attenuated by genetic or pharmacological suppression of glycine receptor function in organotypic slice cultures from mouse cortex (Avila et al., 2013). In this study, the migration speed was not affected by addition of taurine, suggesting that glycine itself acts as endogenous neurotransmitter. In line with this suggestion, the estimated extracellular glycine levels of ~150 nmol/l (Qian et al., 2014) would allow a partial activation of α2 subunit containing receptors with their EC_50_ of ~0.5 µmol/l (Flint et al., 1998; Okabe et al., 2004), while the estimated extracellular taurine concentration of 33 µmol/l (Qian et al., 2014) is most probably ineffective to activate glycine receptors (EC_50_ for taurine ~2.5 mM, Okabe et al., 2004).

In summary, these results indicate that glycine receptors can affect neuronal migration, although these receptors may be relevant only for tangential migration.

## Role of glutamate and GABA in neuronal migration disorders

Given the pivotal role of glutamate and GABA in controlling neuronal migration in the developing cortex, it is not surprising that any modulation in the function of these neurotransmitters during pre- and early postnatal periods may have profound effects on the generation of the cortical architecture. Since neuronal migration is indirectly also controlled by spontaneous network activity, modulation of these transmitter systems may also cause disturbances in early neuronal activity patterns subsequently leading to migration deficits (for review, Kilb et al., 2011).

A direct role of GABA for migration disorders has recently been demonstrated in experimentally induced polymicrogyria. Wang et al. (2014) observed that the accumulation of neurons in the polymicrogyria forming below a neocortical freeze lesion was prevented by the administration of GABA_A_ receptor antagonists *in vivo*. An altered migration caused by excitatory GABA_A_ receptors has also been revealed as a cause for the hippocampal granule cell ectopia observed after febrile seizures (Koyama et al., 2012).

A number of drugs, which are taken by pregnant women for control of psychiatric or neurological disorders (e.g., epilepsy), anesthetics required for surgical operation of pregnant women, or drug abuse (e.g., ethanol consumption during pregnancy) may have profound effects on neuronal migration patterns in the cortex of the unborn child. These drugs often act on glutamate and/or GABA receptors and, when reaching the immature brain, may change the migration pattern of cortical pyramidal cells and GABA interneurons. Anti-epileptic drugs have a wide range of actions (for review Ikonomidou and Turski, 2010): (i) increasing GABAergic action by inhibiting degradation or uptake mechanisms of GABA; (ii) potentiating GABAergic function by acting on GABA_A_ receptor subtypes; (iii) reducing presynaptic glutamate release; (iv) inhibiting glutamate receptor function; and (v) modulating neuronal activity by inhibiting voltage-gated sodium and Ca^2+^ channels. The most potent anti-epileptic drugs are often unspecific and act via several of these mechanisms. In experimental studies, anti-epileptic drugs were administered in clinically relevant doses to pregnant rats during the last week of gestation (for review, Manent et al., 2011). Prenatal exposure to the antiepileptic drugs vigabatrin and valproate, which both increase extracellular GABA levels, induced neuronal migration disorders in the hippocampus and cerebral cortex (Manent et al., 2007). In line with this, the benzodiazepine diazepam, which augments the activation of GABA_A_ receptors, substantially increased the motility rate of migrating GABAergic interneurons (Inada et al., 2011). In contrast the anticonvulsant carbamazepine, which mainly acts on voltage-dependent sodium channels and has only minor effects on GABA receptor function, did not cause major disturbances in neuronal migration (Manent et al., 2007). These data indicate that changes in the extracellular concentration of GABA have a stronger influence on neuronal migration than modifications in spontaneous neuronal activity.

Alcohol exposure during pregnancy is one of the leading causes of mental retardation and several neuroanatomical malformations, including migration disorders such as lissencephaly and cortical heterotopias (for review, Miller, 1992). Ethanol is a drug acting on ionotropic GABA receptors (for review, Lobo and Harris, 2008) and NMDA receptors (for review, Chandrasekar, 2013), which both are key players in the control of neuronal migration. Clinically relevant levels of ethanol substantially impaired migration of cerebellar neurons by attenuating intracellular Ca^2+^ signals (Kumada et al., 2006). In contrast, exposure to relatively low levels of ethanol *in utero* elevated ambient GABA level, enhanced the sensitivity of MGE-derived interneurons to GABA and promoted premature tangential migration into the cortical anlage (Cuzon et al., 2008). A direct link between neuronal migration disorders and glutamate receptor dysfunction has been found in a mouse model for Zellweger disease. Here the migration defect results from a mutation in the NMDA receptor mediated Ca^2+^ mobilization (Gressens et al., 2000).

In summary, exposure of the prenatal human brain to drugs and pharmacological agents acting on glutamate and/or GABA receptors, may have a profound influence on tangential and radial migration. The resulting neuronal migration disorders may be difficult to detect and may result in therapy-resistant neurological and neuropsychiatric disorders.

## Open questions and conclusions

Although the last two decades provided a large amount of experimental data on the role of glutamate and GABA on neuronal migration in the cerebral cortex, a number of questions need to be addressed in order to understand the function of these two classical neurotransmitters and other transmitters in more detail.

1. What is the function of different receptor subunits in neuronal migration? It became recently clear that migrating neurons change during the migration process the expression of their functional receptors, receptor subtypes and subunits. However, the exact role of these developmental changes on the migration process is largely unknown.
2. What are the physiological extracellular concentrations of glutamate, GABA, taurine and glycine in distinct brain regions (e.g., cortical layers) and at different developmental periods? The answer to this question will provide important information to understand tangential and radial migration patterns.
3. What are the roles of other transmitter system beside glutamate and GABA? There are a few reports that other classical neurotransmitters like dopamine or serotonin are also implicated in the control of neuronal migration (e.g., Crandall et al., 2007; Riccio et al., 2011). However, a detailed analysis of the mechanisms of their actions or the identification of other neurotransmitters affecting migration remains open.
4. Which neuronal migration patterns are physiological and which ones are pathophysiological? This question is of major clinical relevance and may be difficult to answer with *in vitro* approaches, where experimental conditions are more or less artificial.
5. When and how does a neuronal migration disorder result in a clinical manifestation of a neurological or neuropsychiatric disease? This is another clinically relevant question and addresses the problem that small structural alterations due to migration disorders may be difficult to detect with conventional imaging techniques.
6. At which time points are the different migration processes respond most sensitive to noxious stimuli? Drugs, hypoxia, inflammation and other noxious conditions in the prenatal brain probably influence the different types and modes of migration in a time-dependent manner. E.g., it is unclear in which developmental period of the prenatal human brain certain drugs have strong impact on radial or tangential migration.
7. What are the (epi-)genetic causes of neuronal migration disorders? Although this field strongly developed over the last decade (for review, LoTurco and Bai, 2006; Guerrini and Parrini, 2010), the influence of genetic and epigenetic factors in distinct migration processes is often still not known.
8. What are the migratory pathways and mechanisms in primates? The vast majority of information on neuronal migration comes from experimental studies on rodents. However, some early events in neocortical development are different between rodents and primates (for review Rakic, 2009). A better understanding of neuronal migration and migration disorders in humans, may require further studies in primates.

In summary, a variety of studies provide substantial evidence that the classical neurotransmitters GABA and glutamate influence neuronal migration and may thus directly contribute to the pathogenesis of neuronal migration disorders. However, the effects of these neurotransmitters are not uniform, but depend on the brain region, identity and maturational state of the migrating neuron and the neurotransmitter receptor subtypes involved. Awareness of the complex interplay between neurotransmitter action and cellular migration processes may help to prevent migration disorders during fetal development.

## Conflict of interest statement

The authors declare that the research was conducted in the absence of any commercial or financial relationships that could be construed as a potential conflict of interest.
